# New hybrid materials based on poly(ethyleneoxide)-grafted polysilazane by hydrosilylation and their anti-fouling activities

**DOI:** 10.3762/bjnano.4.75

**Published:** 2013-10-21

**Authors:** Thi Dieu Hang Nguyen, François-Xavier Perrin, Dinh Lam Nguyen

**Affiliations:** 1 Danang University of Science and Technology, University of Danang, 54 Nguyen Luong Bang, Danang, Vietnam; 2 Institut des Sciences de l’Ingénieur de Toulon - Var (ISITV), Université du Sud Toulon - Var, Av. G. Pompidou, BP 56, 83162 La Valette Cedex, France

**Keywords:** antibacterial, hybrid materials, hydrosilylation, poly(ethyleneoxide), polysilazane

## Abstract

The objective of this work was to develop new coating materials based on poly(ethyleneoxide) (PEO), which was grafted onto polysilazane (PSZ) by hydrosilylation. Three types of PEO with different molecular weights (350, 750, 2000 g/mol) were studied. The kinetics and yields of this reaction have been surveyed by ^1^H and ^13^C NMR spectroscopy. The PEO grafting-density onto PSZ by hydrosilylation increases with a reduction of the S–H/allyl ratio and a decrease of the PEO chain-length. The PEO-*graft*-PSZ (PSZ-PEO) hybrid coatings, which can be used to prevent the adhesion of marine bacteria on surfaces, were applied by moisture curing at room temperature. The anti-adhesion performance, and thus the anti-fouling activity, of the coatings against three marine bacteria species, *Clostridium sp.* SR1, *Neisseria sp.* LC1 and *Neisseria sp.* SC1, was examined. The anti-fouling activity of the coatings depends on the grafting density and the chain length of PEO. The shortest PEO(350 g/mol)-*graft*-PSZ with the highest graft density was found to have the best anti-fouling activity. As the density of grafted PEO(750 g/mol) and PEO(2000 g/mol) chains onto the PSZ surface is approximately equal, the relative effectiveness of these two types of PEO is controlled by the length of the PEO chain. The PEO(2000 g/mol)-*graft*-PSZ coatings are more efficient than the PEO(750 g/mol)-*graft*-PSZ coatings for the bacterial anti-adhesion.

## Introduction

To date, polysilazanes with the general formula –(SiR^1^R^2^–NR^3^)*_n_*–, have been mainly used as ceramic precursors Si/C/N for high-temperature applications [1–3]. The Clariant Company has developed formulations based on polysilazanes that could crosslinked by humid air through hydrolysis and condensation reactions [4–5]. This method was used to prepare coatings of thicknesses from 0.3 to 50 micrometers that have an excellent resistance against corrosion and abrasion because of their exceptional adhesion to a variety of substrates (glass, polycarbonate, aluminium, steel, etc.) combined with a high brightness, a non-stick surface, and the resistance to heat, fire and UV irradiation.

Poly(ethylene oxide) or poly(oxyethylene) (PEO) is a non-toxic polymer, which is used as a surface-modification agent, because it is effective in reducing bio-adhesion, i.e., protein adsorption, or the adhesion of bacteria and cells. The environmentally friendly coatings obtained by the grafting of PEO onto PSZ are a promising way to prevent the deposition of marine fouling materials onto the hulls of ships [6]. Several factors have been hypothesized to explain the anti-fouling property of these surfaces. These include the hydrophilicity [7], the mobility, the large exclusion volume in water and the effect of steric repulsion [8–11]. The effect of the molecular weight and the grafting density of PEO on the anti-fouling capacity has been widely studied [12–14].

Several methods to modify surfaces with PEO chains were investigated. These include the covalent grafting [15–16], the chemical adsorption [17], the formation of self-assembled monolayers [18–19], plasma treatment [12,20] or the use of supercritical CO_2_ [21–22]. Kingshott et al. have demonstrated that the grafting of PEO onto the substrates by covalent bonding is necessary for an anti-fouling activity [23]. Herein, we propose a new synthetic strategy to prepare hybrid materials based on polysilazane (PSZ), which have an enhanced resistance against bacteria adhesion, through the incorporation of allyl PEO monomethyl ether by covalent grafting.

## Experimental

**Figure 1:**
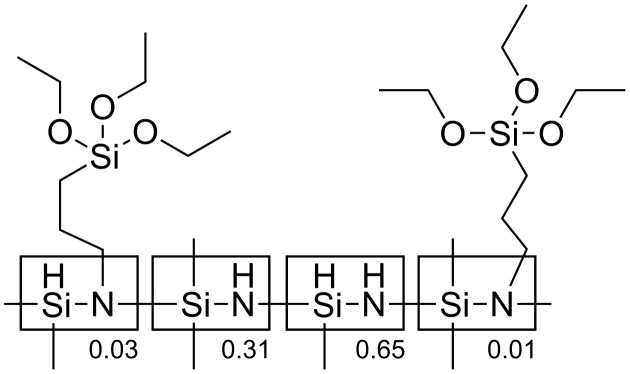
Molecular structure and distribution of the functional groups of the polysilazane precursor provided by the Clariant Company.

**Materials.** Monomethoxy poly(ethylene oxide) glycol (MPEG) with average molecular weights of 350 g/mol, 750 g/mol and 2000 g/mol and Karstedt’s catalyst were purchased from Sigma-Aldrich. All three types of MPEG were dried under vacuum before usage. The polysilazane precursor, polylmethyhydrosilazane containing triethoxysilanes, was provided by the Clariant Company. The molecular structure of the polysilazane precursor is described on Figure 1. PSZ was used as received without any further purification. Allyl bromide (allyl-Br) purchased from Acros was distilled under a nitrogen atmosphere in the dark and subsequently kept in darkness before usage. Allyl-Br is sensitive to light and may polymerize on exposure to light.

**NMR.****^1^H and ^13^C NMR spectra were obtained on a Bruker FT-NMR 400 MHz spectrometer.

**Protocol of the marine bacterial adhesion.** Three marine bacteria species, bacillus *Clostridium sp.* SR1, micrococcus *Neisseria sp.* LC1 and micrococcus *Neisseria sp.* SC1, were used for the adhesion studies. The culture media was Vätäänen nine-salt solution (VNSS). The PSZ and PSZ-PEO coatings, which were moisture-cured at ambient conditions, were previously sterilized in methanol, immersed in VNSS that contained the bacteria and were finally incubated at 30 °C for 24 h. Then coatings were rinsed in sterilized phosphate buffered saline to remove the non-adhered bacteria. The adhered bacteria were removed from the surfaces using an ultrasonic cleaner for 5 min with sterilized synthetic sea water. The number of adhered bacteria (CFU/cm^2^) was determined by using a Colony Counter BZG30.

**Scheme 1:**
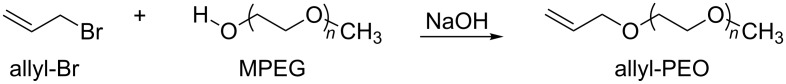
Equation of the allyl-PEO synthesis reaction.

**Synthesis of allyl-PEO.** Allyl-terminated monomethyl poly(ethylene oxide) (allyl-PEO) ethers were synthesized via the reaction of MPEG with an excess amount of allyl-Br [24]. The equation of this synthesis reaction is described in Scheme 1.

**Scheme 2:**
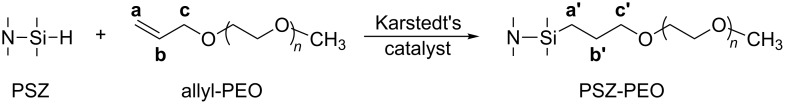
Principle of hydrosilylation between the Si–H group of PSZ and the C=C bond of the allyl-PEO.

**Synthesis of PSZ-PEO.** Grafting of allyl-PEO molecules onto the polysilazane (PSZ) chain was performed by using a hydrosilylation reaction between the Si–H group of PSZ and the terminal C=C bond of the allyl-PEO with an excess amount of PSZ in presence of Karstedt’s catalyst (Scheme 2). We define that **a**, **b**, **c** are the different protons and carbon atoms on the allyl branch of the PEO molecule, whereas **a′**, **b′**, **c′** are the different protons and carbon atoms of the same branch *after* being grafted onto the PZS chain.

The most favorable conditions for the grafting of allyl-PEO chains on PSZ are the following: a temperature of 80 °C and a molar ratio of Pt/allyl of 3 × 10^−3^ under argon atmosphere. Different Si–H/allyl molar ratios of 10, 16.5 and 26.5, respectively, were investigated for all three types of allyl-PEO. The grafted products are symbolized as PSZ-PEO**X**-**Y** with **X** being the molecular weight of MPEG and **Y** being the Si–H/allyl molar ratio.

## Results and Discussion

### Grafting of allyl-PEO molecules onto the PSZ chain

**Figure 2:**
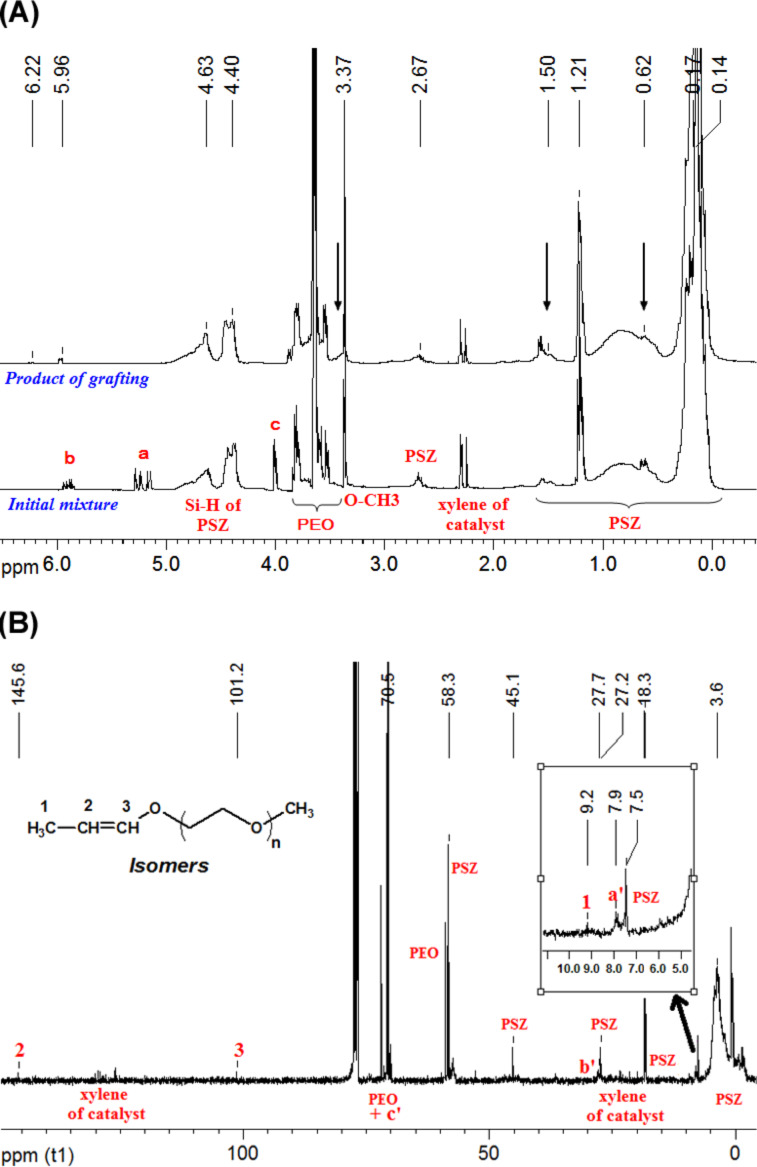
Identification of the grafting of PEO350 molecules onto PSZ chain. (A) Comparison of ^1^H NMR spectra of initial mixture and grafting product. (B) ^13^C NMR spectrum of grafting product.

The kinetics and yields of the grafting reactions have been surveyed by ^1^H and ^13^C NMR spectroscopy. Figure 2 shows the ^1^H NMR and ^13^C NMR spectra of the mixture of the reactants and of the grafting products. In Figure 1 and in Scheme 2, it is found that the structure of the grafted group ≡Si–CH_2_–CH_2_–CH_2_–O– of PSZ-PEO is very close to that of the group ≡Si–CH_2_–CH_2_–CH_2_–N– of the PSZ precursor. In the ^1^H NMR spectra, the resonance peaks of the protons ***H*_a_*_'_*** and ***H*_b_*_'_*** of PSZ-PEO therefore appear at chemical shifts very similar to those of the protons ≡Si–C***H*_2_**–CH_2_– (0.62 ppm) and ≡Si–CH_2_–C***H*_2_**– (1.5 ppm) of PSZ, respectively. Likewise, the chemical shift of the proton ***H*_c_*_'_*** of PSZ-PEO is very close to that of protons –O–C***H*_3_** and –O–C***H*_2_**–C***H*_2_**–O– of PEO, situated at around 3.4 ppm (Figure 2A). The same difficulties in identifying the grafted PEO are encountered during the analysis of the ^13^C NMR spectra. This is because the ^13^C NMR chemical shifts of the carbon atoms at positions ***C*_a_**_’_ and ≡Si–***C***H**_2_**–CH_2_–, ***C*_b_**_’_ and ≡Si–CH_2_–***C***H**_2_**–, ***C***_c’_ and –O–***C***H_2_–***C***H_2_–O– of PEO are very close, as shown in Figure 2B. In order to circumvent theses complications, the kinetic investigation of the hydrosilylation reaction was carried out by following the decrease of the intensity of the allyl-proton resonances in the ^1^H NMR spectra.

**Figure 3:**
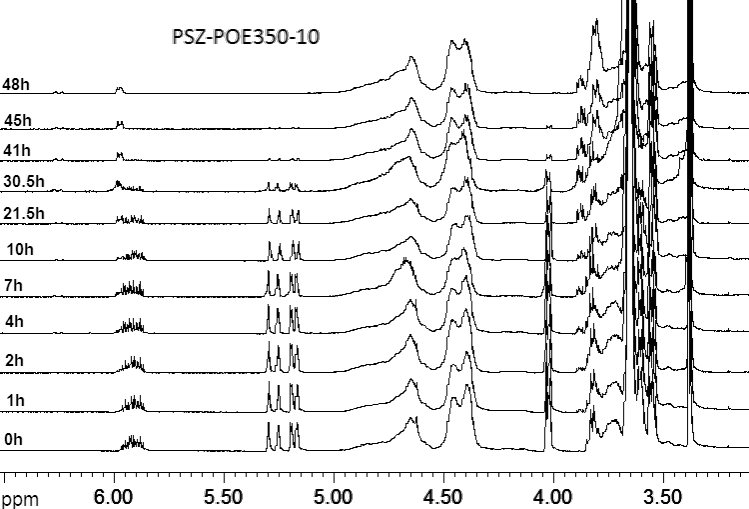
Investigation of grafting allyl-PEO350 onto the PSZ chain by ^1^H NMR (SiH/allyl = 10).

The evolution of the ^1^H NMR spectra of the reactant mixture over the reaction time is shown in Figure 3. These results verify that the intensities of the ^1^H MNR peaks attributed to the allyl group (–C***H*** = at 5.87ppm, C***H***_2_ = at 5.22ppm and –**C*H*_2_**− at 3.99ppm) [24] decrease continuously. This diminution can be used to confirm the participation of the allyl group in the hydrosilylation during the grafting of allyl-PEO onto the PSZ chain.

**Figure 4:**
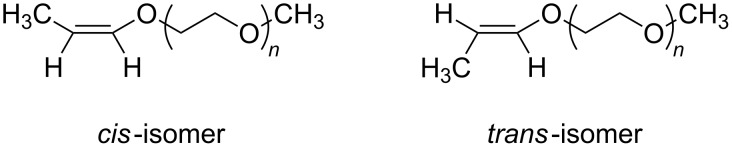
Chemical structure of *cis*- and *trans*-isomers of allyl-PEO.

After few hours of the grafting reaction, we recognized the appearance of two new ^1^H NMR peaks at chemical shifts of 5.9 and 6.2 ppm. The intensities of two new peaks increase with reaction time. These two peaks have been verified to match the =C***H***− protons of the *cis*- and *trans*-isomers, respectively, of the propenyl ether group, which results from the isomerization of allyl-PEO [25] (Figure 4). In the ^13^C NMR spectrum (Figure 2), the position of the propenyl ether group has been recognized in the region of low fields (***C***H_3_− at 9.2ppm, −***C***H= at 145.6ppm and =***C***H−O at 101.2ppm). The isomerization of the allyl-PEO was therefore considered as a secondary reaction that takes place simultaneously to the hydrosilylation. The optimum conditions for the grafting of PEO were defined to reach a high selectivity toward the hydrosilylation in comparison to the isomerisation.

**Figure 5:**
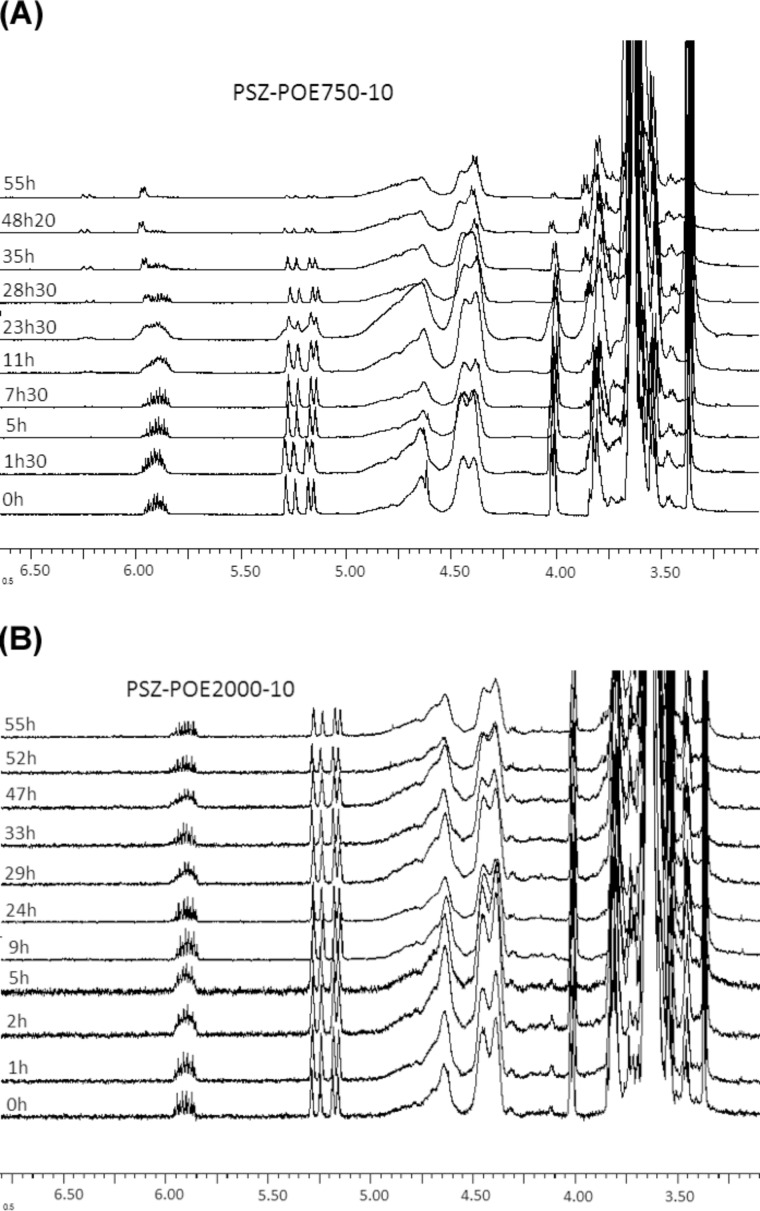
Investigation of grafting allyl-PEO750 (A) and allyl-PEO2000 (B) onto the PSZ chain by ^1^H NMR (SiH/allyl = 10).

It was observed that the conversion of allyl-PEO350-10 was 100% after 48 h. In contrast, allyl-PEO750 and allyl-PEO2000 were not converted completely in any ratio of SiH/allyl after 55 h of grafting (Figure 5 and below in Table 1). The amount of non-reacted allyl-PEO2000 can be seen very clearly in the ^1^H NMR spectra. This means, the longer the molecule the more difficult is its grafting. Therefore, the products of the grafting of allyl-PEO750 and allyl-PEO2000 are mixtures of PSZ-PEO, PSZ surplus, propenyl ether isomers and unreacted allyl-PEO.

The decrease in the ^1^H NMR signal of C***H*_2_**= was also used to evaluate the conversion of allyl-PEO during grafting. The total conversion of allyl-PEO (%C_AP,total_), the conversion of Allyl-PEO associated with isomerization (%C_AP,iso_) and the conversion of allyl-PEO associated with hydrosilylation (%C_AP,hydro_) were calculated according to the following equations:

[1]
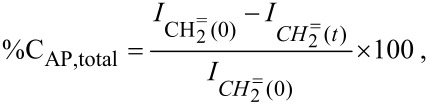


[2]
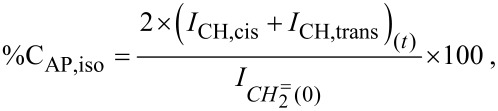


[3]
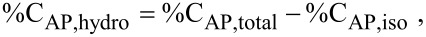


in which I_CH2=(0)_ is the integral of the C***H_2_***= resonance in the ^1^H-NMR spectrum of the initial mixture and I_CH2=(_*_t_*_)_, I_CH,cis(_*_t_*_)_ and I_CH,trans(_*_t_*_)_ are the integrals of the C***H_2_***=, *cis*-C***H***=, and *trans*-C***H***= resonances at time *t*, respectively.

The density (δ) of grafted PEO molecules onto the PSZ chain expressed by the amount of PEO attaching to 10 equiv of initial S−H has been calculated according to

[4]
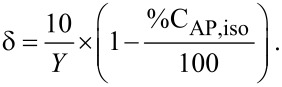


The conversions of all three types of allyl-PEO in the different reactions during grafting as well as the density of grafted allyl-PEO molecules onto the PSZ chain are listed in Table 1. It should be noted that the degree of hydrosilylation increases when increasing the Si–H/allyl ratio and decreasing the molecular weight of PEO. Contrary to this, the degree of isomerization reduces significantly when increasing the Si–H/allyl ratio. In particular, when Si–H/allyl = 26.5, the *trans*-isomer could not be identified for both allyl-PEO750 and allyl-PEO2000 in their ^1^H NMR spectra.

**Table 1:** Conversion of allyl-PEO and density of the grafted PEO chains onto PSZ calculated from ^1^H NMR spectra.

X = *M*_allyl-PEO_ (g/mol)	Y = SiH/allyl	*t* (h)	%C_AP,total_	%C_AP,hydro_	%C_AP,iso_	δ (mol PEO grafted/10 mol of Si–H)

350	10	48	100	71.78	28.22	0.72
	16.5	40	100	83.63	16.37	0.51
	26.5	30	100	91.84	8.16	0.35

750	10	55	96.72	62.84	33.88	0.63
	16.5	55	91.60	68.02	23.59	0.41
	26.5	55	97.35	88.86	8.49	0.34

2000	10	55	80.15	59.95	20.20	0.60
	16.5	55	72.65	61.96	10.69	0.38
	26.5	55	86.61	78.07	8.54	0.29

Table 1 clearly shows that the density of the grafted PEO onto PSZ chain increases when the initial S–H/allyl ratio and the PEO molecular weight decrease. To increase the Si–H/allyl ratio means that the concentration of allyl-PEO molecules will become low. So in this case, the density of grafted PEO was reduced despite increasing the degree of hydrosilylation.

### Marine bacterial adhesion on modified surfaces

**Figure 6:**
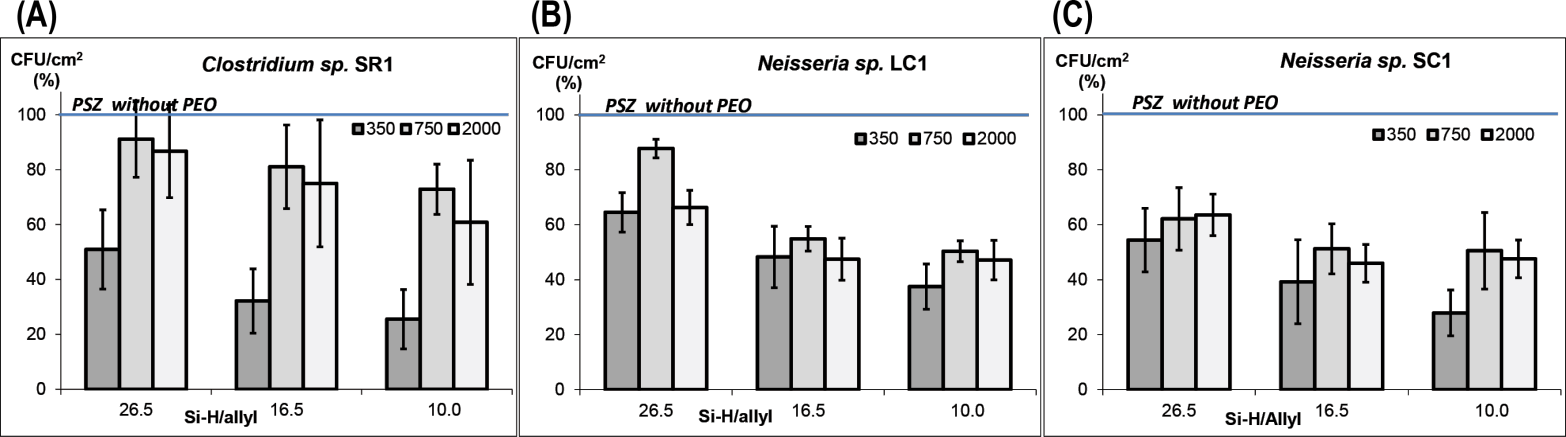
Marine bacteria adhesion on PSZ-PEO surfaces (mean ± SD, 100% corresponds to 13 × 10^2^ CFU/cm^2^ for *Clostridium sp.* SR1 (A); 8 × 10^3^CFU/cm^2^ for *Neisseria sp.* LC1 (B) and 11 × 10^3^CFU/cm^2^ for *Neisseria sp.* SC1 (C)).

Figure 6 shows the influence of the length of the grafted PEO and of the Si–H/allyl ratio on the bacterial adhesion in VNSS media. Compared to the unmodified PSZ coating, all PSZ-PEO surfaces exhibit lower levels of bacteria adhesion. The inhibition efficiency varies inversely with the ratio of Si–H/allyl. It is remarkable that the PSZ-PEO350-10 surface shows the best inhibitory properties and leads to a reduction in the amount of adhered bacteria of approximately 70% for all three types of bacteria when compared to the pure PSZ coating. The anti-fouling activities of PSZ-PEO showed an increase in the sequence PEO750 < PEO2000 < PEO350. This result may seem to disagree with several previous studies showing that a PEO2000 chain is most effective against bacterial adhesion [12,26–27]. However, all PSZ-PEO350 coatings have a PEO grafting density that is higher than that of PSZ-PEO750 and PSZ-PEO2000 at the same Si–H/allyl ratio (see Table 1). The higher PEO grafting density of PSZ-PEO350 could explain its higher anti-fouling activity. These results are also in accordance with the works of Jeon et al. [9–10]. Based on a surface–protein interaction model these authors demonstrated that the surface density of the PEO chains have a greater effect than the length of the PEO chains in inhibiting bacterial adhesion. As the grafting density of PEO750 and PEO2000 onto PSZ is approximately equivalent, the effectiveness of these two types of hybrid materials is controlled by the chain length of PEO. Consequently, the PSZ-PEO2000 surfaces are more efficient than the PSZ-PEO750 surfaces in regarding to the anti-fouling activity.

## Conclusion

The grafting of allyl-PEO molecules onto a PSZ chain in presence of Karstedt’s catalyst has been successfully realized by hydrosilylation. The structure of the grafted products have been identified by ^1^H NMR and ^13^C NMR. After a few hours of the grafting reaction, there are the appearances of *trans*- and *cis*-isomers of allyl-PEO molecules. Apparently, isomerization occurs simultaneously to the hydrosilylation. The grafting density of PEO onto the PSZ chain increases with a reduction of the S–H/allyl ratio and a decrease of the chain length of PEO. At a molar ratio of Si–H/allyl = 10 for grafting allyl-PEO350 molecules, there is 0.72 chains of allyl-per ten Si–H groups of the PSZ chain. While at a molar ratio of Si–H/allyl = 26.5 for grafting allyl-PEO2000 molecules, there is 0.29 chains of allyl-PEO per ten Si–H groups of the PSZ chain.

The bacterial adhesion inhibition depends on the grafting density and the chain length of PEO. The role of the grafting density is more important than that of the chain length. The PSZ-PEO350 coatings prepared at a ratio SiH/allyl = 10 with the highest grafting density show the best anti-fouling activity for all three studied marine bacteria. While the densities of PEO750 and PEO2000 chains on the PSZ surface are practically equal, the PSZ-PEO2000 coatings are more efficient in anti-fouling than those with PSZ-PEO750. It is indispensable to find the optimal conditions for grafting allyl-PEO2000 on the PSZ chain in order to improve the degree of hydrosilylation and to increase the density of grafted allyl-PEO2000 molecules in PSZ-PEO anti-fouling surfaces.
